# Supplementary material for: Guanidine hydrochloride reactivates an ancient septin hetero-oligomer assembly pathway in budding yeast
Source: eLife. 2020 Jan 28;9:e54355. doi: 10.7554/eLife.54355 (PMC7056273; doi:10.7554/eLife.54355)
Supplement: Supplementary file 1. [file elife-54355-supp1.docx]

| **Key Resources Table** | | | | |
| --- | --- | --- | --- | --- |
| **Reagent type (species) or resource** | **Designation** | **Source or reference** | **Identifiers** | **Additional information** |
| gene (*Saccharomyces cerevisiae*) | *CDC3* |  | NCBI Gene 851024 |  |
| gene (*Saccharomyces cerevisiae*) | *CDC10* |  | NCBI Gene 850358 |  |
| gene (*Ashbya gossypii*) | *CDC10* |  | NCBI Gene 28725232 |  |
| gene (*Saccharomyces cerevisiae*) | *CDC11* |  | NCBI Gene 853539 |  |
| gene (*Saccharomyces cerevisiae*) | *CDC12* |  | NCBI Gene 856507 |  |
| gene (*Saccharomyces cerevisiae*) | *SHS1* |  | NCBI Gene 851373 |  |
| gene (*Ashbya gossypii*) | *SHS1* |  | NCBI Gene 4618868 |  |
| gene (*Saccharomyces cerevisiae*) | *DPL1* |  | NCBI Gene 851888 |  |
| gene (*Aspergillus nidulans*) | *aspB* |  | NCBI Gene 2870475 |  |
| genetic reagent (*S. cerevisiae*) | *cdc10(D182N)-GFP::URA3 hsp78∆::kanMX* | This study |  | Yeast strain created in M. McMurray lab by appropriate crosses from strains maintained in McMurray lab. |
| genetic reagent (*S. cerevisiae*) | *cdc10(D182N)-GFP::URA3 hsp104∆::kanMX* | This study |  | Yeast strain created in M. McMurray lab by appropriate crosses from strains maintained in McMurray lab. |
| genetic reagent (*S. cerevisiae*) | *cdc10(D182N)-GFP::URA3 hsp78∆::kanMX hsp104∆::kanMX* | This study |  | Yeast strain created in M. McMurray lab by appropriate crosses from strains maintained in McMurray lab. |
| genetic reagent (*A. gossypii*) | Ag135 (A.g., also called AgHPH14) *cdc10∆::GEN1 SHS1-GFP–NAT1* | (Helfer & Gladfelter, 2006) |  | Fungal strain maintained in A. Gladfelter lab |
| genetic reagent (*S. cerevisiae*) | BY4741 (*MAT****a*** *his3*∆*1 leu2*∆*0 ura3*∆*0 met15*∆*0*) | (Brachmann et al., 1998) | ATCC® 201388™ | Yeast strain maintained at American Type Culture Collection |
| genetic reagent (*S. cerevisiae*) | BY4742 (*MAT*α *his3*∆*1 leu2∆0 ura3*∆*0 lys2*∆*0*) | (Brachmann et al., 1998) | ATCC® 201389™ | Yeast strain maintained at American Type Culture Collection |
| genetic reagent (*S. cerevisiae*) | CBY05110 (BY4741 “*cdc12-1” cdc12(G247E)::KanMX*) | (Li et al., 2011) |  | Yeast strain maintained in C. Boone lab |
| genetic reagent (*S. cerevisiae*) | CBY06417 (BY4741 “*cdc10-1” cdc10(D182N)::KanMX*) | (Li et al., 2011) |  | Yeast strain maintained in C. Boone lab |
| genetic reagent (*S. cerevisiae*) | CBY06420 (BY4741 “*cdc10-2” cdc10(G100E)::KanMX*) | (Li et al., 2011) |  | Yeast strain maintained in C. Boone lab |
| genetic reagent (*S. cerevisiae*) | CBY06424 (BY4741 “*cdc10-5” cdc10(G44D)::KanMX*) | (Li et al., 2011) |  | Yeast strain maintained in C. Boone lab |
| genetic reagent (*S. cerevisiae*) | CBY06427 (BY4741 *cdc11(G29D)::KanMX*) | (Li et al., 2011) |  | Yeast strain maintained in C. Boone lab |
| genetic reagent (*S. cerevisiae*) | CBY07236 (BY4741 *“cdc3-3” cdc3(G365R)::KanMX*) | (Li et al., 2011) |  | Yeast strain maintained in C. Boone lab |
| genetic reagent (*S. cerevisiae*) | CBY08756 (BY4741 “*cdc11-1” cdc11(G32E I142T)::KanMX*) | (Li et al., 2011) |  | Yeast strain maintained in C. Boone lab |
| genetic reagent (*S. cerevisiae*) | JTY3985 (BY4741 *CDC10-GFP::URA3*) | (Johnson et al., 2015) |  | Yeast strain maintained in M. McMurray lab |
| genetic reagent (*S. cerevisiae*) | JTY3986 (BY4741 *cdc10(D182N)-GFP::URA3*) | (Johnson et al., 2015) |  | Yeast strain maintained in M. McMurray lab |
| genetic reagent (*S. cerevisiae*) | JTY3992 (BY4741 *CDC10-mCherry::KanMX*) | (McMurray & Thorner, 2008) |  | Yeast strain maintained in M. McMurray lab |
| genetic reagent (*S. cerevisiae*) | JTY3993 (BY4742 *CDC10-mCherry::KanMX*) | (McMurray & Thorner, 2008) |  | Yeast strain maintained in M. McMurray lab |
| genetic reagent (*S. cerevisiae*) | BY4742 *hsp104∆::kanMX*) | (Winzeler et al., 1999) | YSC6272-201918417 | Yeast strain maintained at Horizon Discovery |
| genetic reagent (*S. cerevisiae*) | BY4741 *hsp104∆::KanMX*) | (Winzeler et al., 1999) | ATCC® 4001514™ | Yeast strain maintained at American Type Culture Collection |
| genetic reagent (*S. cerevisiae*) | JTY4020 (BY4743 *CDC10-mCherry::kanMX/cdc10(D182N)-GFP::URA3*) | This study |  | Yeast strain maintained in M. McMurray lab. Made by mating JTY3993 with JTY3985. |
| genetic reagent (*S. cerevisiae*) | JTY4203 (*cdc10∆::kanMX* [*CDC10 URA3*]) | (McMurray et al., 2011) |  | Yeast strain maintained in M. McMurray lab |
| genetic reagent (*S. cerevisiae*) | JTY5104 (*cdc3∆ cdc10∆* [*CDC3(W364A) LYS2*] [*CDC10 URA3*]) | (McMurray et al., 2011) |  | Yeast strain maintained in M. McMurray lab |
| genetic reagent (*S. cerevisiae*) | JTY5397 (BY4741 *CDC10-mCherry::kanMX SHS1-GFP::HIS3MX6*) | (Weems & McMurray, 2017) |  | Yeast strain maintained in M. McMurray lab |
| genetic reagent (*S. cerevisiae*) | MMY0110 (*cdc3(D210G) cdc10(D182N)*) | (Weems et al., 2014) |  | Yeast strain maintained in M. McMurray lab |
| genetic reagent (*S. cerevisiae*) | MMY0130 (*cdc3(D210G) cdc10(D182N)*) | (Weems et al., 2014) |  | Yeast strain maintained in M. McMurray lab |
| genetic reagent (*S. cerevisiae*) | MMY0131 (*cdc3(D210G)*) | (Weems et al., 2014) |  | Yeast strain maintained in M. McMurray lab |
| genetic reagent (*S. cerevisiae*) | BY4742 *hsp78∆::kanMX* | (Winzeler et al., 1999) | ATCC® 4013617™ | Yeast strain maintained at American Type Culture Collection |
| genetic reagent (*S. cerevisiae*) | MMY0343 (BY4741 *CDC10-mCherry::kanMX SHS1-GFP::HIS3MX6 cdc3(95-341)::aspB(6-234)*) | This study |  | Yeast strain maintained in M. McMurray lab. Derived from JTY5397. See Methods section for details. Following confirmation of the integration via PCR and sequencing, spontaneous loss of plasmid pEM-CDC3-CRISPR1 was selected using 5-FOA. |
| genetic reagent (*S. cerevisiae*) | MMY0350 (BY4741 *cdc10(Q265H)-mCherry::kanMX SHS1-GFP::HIS3MX6 cdc3::aspB(6-234)*) | This study |  | Yeast strain maintained in M. McMurray lab. Derived from JTY5397. See Methods section for details. Following confirmation of the integration via PCR and sequencing, spontaneous loss of plasmid pEM-CDC3-CRISPR1 was selected using 5-FOA. |
| genetic reagent (*S. cerevisiae*) | MMY0361 (BY4741 *CDC10-mCherry::kanMX SHS1-GFP::HIS3MX6 CDC3(P127E D128S K181T T302R Q306D)*) | This study |  | Yeast strain maintained in M. McMurray lab. Derived from JTY5397. See Methods section for details. Following confirmation of the integration via PCR and sequencing, spontaneous loss of plasmid pEM-CDC3-CRISPR1 was selected using 5-FOA. |
| genetic reagent (*S. cerevisiae*) | MMY0362 (BY4741 *CDC10-mCherry::kanMX SHS1-GFP::HIS3MX6 CDC3”recoded”*) | This study |  | Yeast strain maintained in M. McMurray lab. Derived from JTY5397. See Methods section for details. Following confirmation of the integration via PCR and sequencing, spontaneous loss of plasmid pEM-CDC3-CRISPR1 was selected using 5-FOA. |
| genetic reagent (*S. cerevisiae*) | MMY0363 (BY4741 *CDC10-mCherry::kanMX SHS1-GFP::HIS3MX6 CDC3(T302R Q306D)*) | This study |  | Yeast strain maintained in M. McMurray lab. Derived from JTY5397. See Methods section for details. Following confirmation of the integration via PCR and sequencing, spontaneous loss of plasmid pEM-CDC3-CRISPR1 was selected using 5-FOA. |
| genetic reagent (*S. cerevisiae*) | Byk739 (*ade2∆::HIS3MX/ade2∆::HIS3MX cdc10∆::URA3/CDC10 dpl1∆::natMX/DPL1 psd2∆::kanMX/PSD2*) | (Michel et al., 2017) |  | Yeast strain maintained in B. Kornmann lab |
| genetic reagent (*S. cerevisiae*) | MMY0370 (*ade2∆::HIS3MX cdc10∆::URA3 dpl1∆::natMX PSD2*) | This study |  | Yeast strain maintained in M. McMurray lab. Made by sporulation of Byk739, dissection of asci, germination of spores, and analysis of markers to identify a spore clone of the appropriate genotype. |
| sequenced-based reagent | Cdc3AspBfw | This study | PCR primers | GGTATGGGCATCACCTCCTCTCAGAGCGAAAAGGGTCAAGTTCTGCCTGACCAACCGGAGATTAAGTTCATTCGCCGGAAGCTAACCGGC |
| sequenced-based reagent | Cdc3AspBextend1 | This study | PCR primers | GGAACGGCCTCTCACTTGATTACCGCTATAATTTTCCACGATGTCGTTGGAACCAATAACGGCAAAAGGAACCTTCGACAT |
| sequenced-based reagent | Cdc3GTPase_extend2 | This study | PCR primers | TTTCAAAAGGTTGAAATCAGAGTGGTTGTCATTGTCCACTTCGATCACCCCCCATGGATAGGAACGGCCTCTCACTTG |
| sequenced-based reagent | 5’cdc3fw | This study | PCR primers | AACTGAACGATTACATCGGC |
| sequenced-based reagent | 3’cdc3re | This study | PCR primers | TGTTTGAAATTTTTATATGTC |
| sequenced-based reagent | 5’cdc10fw | This study | PCR primers | CACGGTTACTACAAGCACTCT |
| sequenced-based reagent | 3’cdc10re | This study | PCR primers | TCACCACCATTCTTATGAGA |
| sequenced-based reagent | cdc10midfw | This study | PCR primers | CCAAATGGAAAGGAGTTGAGC |
| sequenced-based reagent | CDC3GTPaserecode | This study | Synthetic gene block | ATTCGTCGTCAAATCAATGGTTATGTAGGGTTTGCTAATTTGCCCAAGCAATGGCACAGAAGGTCCATAAAGAACGGATTCAGTTTCAATCTCCTATGTGTCGGCGAGAGCGGTATAGGTAAGACAACATTAATGAAAACATTATTTAATAACGATGATATTGAAGCCAATTTAGTCAAAGATTACGAAGAGGAACTTGCTAATGATCAGGAGGAAGAGGAGGGACAGGGCGAGGGACACGAGAACCAGTCACAAGAGCAAAGGCACACTGTCAAAATCAAGTCATATGAGTCGGTTATTGAAGAAAATGGTGTTAAACTGAATTTGAACGTGATCGATACTGAAGGATTTGGTGATTTTTTAAATAATGATCAAAAATCATGGGACCCGATCATTAAGGAGATTGACTCACGTTTTGACCAATACTTAGATGCGGAGAATAAAATCAATAGACATTCTATAAACGACAAAAGAATCCACGCATGTCTATATTTCATTGAGCCTACAGGACACTACTTAAAGCCGCTGGACCTTAAGTTTATGCAGTCAGTATATGAGAAATGTAATTTGATTCCTGTAATTGCTAAATCAGACATCTTAACAGATGAAGAGATATTATCCTTCAAGAAGAGAATTATGAACGATTTAATACAATCAAATATAGAGCTTTTCAAACCACCAATATACTCCAACGATGACGCCGAAAATAGCCACTTGTCCGAAAGACTATTTTCCTCCTTACCTTACGCAGTTATTGGTTCAAATGACATCGTGGAAAATTATAGCGGTAATCAAGTGAGAGGCCGTTCCTATCCATGGGGGGTGATCGAAGTGGACAATGACAACCACTCTGATTTCAACCTTTTGAAA |
| sequenced-based reagent | CDC3recode | This study | Synthetic gene block | ATTCGTCGTCAAATCAATGGTTATGTAGGGTTTGCTAATTTGCCCAAGCAATGGCACAGAAGGTCCATAAAGAACGGATTCAGTTTCAATCTCCTATGTGTCGGCCCTGATGGTATAGGTAAGACAACATTAATGAAAACATTATTTAATAACGATGATATTGAAGCCAATTTAGTCAAAGATTACGAAGAGGAACTTGCTAATGATCAGGAGGAAGAGGAGGGACAGGGCGAGGGACACGAGAACCAGTCACAAGAGCAAAGGCACAAGGTCAAAATCAAGTCATATGAGTCGGTTATTGAAGAAAATGGTGTTAAACTGAATTTGAACGTGATCGATACTGAAGGATTTGGTGATTTTTTAAATAATGATCAAAAATCATGGGACCCGATCATTAAGGAGATTGACTCACGTTTTGACCAATACTTAGATGCGGAGAATAAAATCAATAGACATTCTATAAACGACAAAAGAATCCACGCATGTCTATATTTCATTGAGCCTACAGGACACTACTTAAAGCCGCTGGACCTTAAGTTTATGCAGTCAGTATATGAGAAATGTAATTTGATTCCTGTAATTGCTAAATCAGACATCTTAACAGATGAAGAGATATTATCCTTCAAGAAGACCATTATGAACCAGTTAATACAATCAAATATAGAGCTTTTCAAACCACCAATATACTCCAACGATGACGCCGAAAATAGCCACTTGTCCGAAAGACTATTTTCCTCCTTACCTTACGCAGTTATTGGTTCAAATGACATCGTGGAAAATTATAGCGGTAATCAAGTGAGAGGCCGTTCCTATCCATGGGGGGTGATCGAAGTGGACAATGACAACCACTCTGATTTCAACCTTTTGAAA |
| sequenced-based reagent | G1KTTRQDfw | This study | PCR primers | ATTCGTCGTCAAATCAATGGTTATG |
| sequenced-based reagent | Cdc3recodere | This study | PCR primers | TTTCAAAAGGTTGAAATCAGAGTGG |
| strain, strain background (*Escherichia coli*) | BL21(DE3) | Sigma-Aldrich | CMC0016 | Electrocompetent cells |
| recombinant DNA reagent | pEM-CDC3-CRISPR1 (plasmid) | Ed Marcotte lab |  | encodes Cas9 and guide RNA targeting CDC3 locus |
| recombinant DNA reagent | pRL10 (plasmid) | Michelle Momany lab |  | encodes *aspB* gene |
| recombinant DNA reagent | pFM831 (plasmid) | Marian Farkasovsky lab(Sirajuddin et al., 2009) |  | low-copy yeast plasmid encoding wild-type Cdc3 with *LEU2* marker |
| recombinant DNA reagent | pML109 (plasmid) | (Nagaraj et al., 2008) |  | low-copy yeast plasmid encoding wild-type Cdc3 C-terminally tagged with GFP derivative eCitrine, with *LEU2* marker |
| recombinant DNA reagent | YCpHLcdc3(T302R)-GFP (plasmid) | This study |  | derivative of pML109 with T302R mutation introduced into Cdc3 via site-directed mutagenesis performed by Keyclone Technologies (San Marcos, CA) |
| recombinant DNA reagent | YCpHLcdc3(T302V)-GFP (plasmid) | This study |  | derivative of pML109 with T302V mutation introduced into Cdc3 via site-directed mutagenesis performed by Keyclone Technologies (San Marcos, CA) |
| recombinant DNA reagent | YCpK-Cdc10-1-GFP (plasmid) | (Johnson et al., 2015) |  | low-copy yeast plasmid, encodes *cdc10(D182N)-GFP*, marked with *kanMX* |
| recombinant DNA reagent | pMVB121 (plasmid) | (Versele & Thorner, 2004) |  | *E. coli* plasmid for T7-driven expression of 6xHis-tagged Cdc12 |
| recombinant DNA reagent | pMVB133 (plasmid) | (Versele et al., 2004) |  | *E. coli* plasmid for T7-driven co-expression of Cdc3 and Cdc11 |
| Software, algorithm | ImageJ/FIJI | (Schneider et al., 2012) | RRID:SCR_002285 | Image analysis |
| software, algorithm | “Get plot profile Min-Max” macro for Image J/FIJI |  |  | Automates extraction of peak heights from measurement of septin ring fluorescence. Runs as macro in ImageJ, available at https://github.com/michael-mcmurray/eLife2020software/blob/master/Get%20Plot%20Profile%20Min-Max.txt |
| software, algorithm | Autodoc Vina via Chimera 1.11 | [(Trott & Olson, 2010), http://www.rbvi.ucsf.edu/chimera, (Pettersen et al., 2004)](http://www.rbvi.ucsf.edu/chimera) | **RRID:SCR_011958** | in silico docking software |
| software, algorithm | I-TASSER server | http://zhanglab.ccmb.med.umich.edu/I-TASSER/ (Zhang, 2008) | RRID:SCR_014627 | protein structure prediction software/server |
| software, algorithm | Prism 8.0 | GraphPad | RRID:SCR_002798 | preparation of plots |
| software, algorithm | Phylogeny.fr platform online | http://www.phylogeny.fr/ (Dereeper et al., 2008) | RRID:SCR_010266 | preparation of phylogenetic tree |
| software, algorithm | DABEST (data analysis with bootstrap-coupled estimation) | [http://www.estimationstats.com (Ho et al., 2019)](http://www.estimationstats.com/) |  | preparation of plots and determination of effect sizes and associated distributions |
| Software, algorithm | EMAN software suite | (Ludtke et al., 1999) | RRID:SCR_016867 | two-dimensional image processing for single-particle EM |
| Software, algorithm | SPIDER | (Frank et al., 1996) |  | two-dimensional image processing for single-particle EM |
| chemical compound, drug | guanidine hydrochloride | Sigma-Aldrich | G4505 |  |
| chemical compound, drug | urea | Bio-Rad | 1610730 |  |
| chemical compound, drug | arginine hydrochloride | Sigma-Aldrich | A5131 |  |
| chemical compound, drug | aminoguanidine hydrochloride | Santa Cruz Biotechnology | sc-202931 |  |
| chemical compound, drug | N-ethylguanidine hydrochloride | Santa Cruz Biotechnology | sc-269833 |  |
| antibody | anti-Cdc11 primary antibody, rabbit polyclonal | Santa Cruz Biotechnology | sc-7170, RRID:AB_671797 | IF (1:5000) |
| antibody | Alexa-Fluor 488-labeled goat anti-rabbit secondary antibody | Biotium | 20012, RRID:AB_10853801 | IF (1:1000) |
